# Hippocampal subfield volumes are nonspecifically reduced in premature‐born adults

**DOI:** 10.1002/hbm.25187

**Published:** 2020-08-26

**Authors:** Dennis M. Hedderich, Mihai Avram, Aurore Menegaux, Rachel Nuttall, Juliana Zimmermann, Sebastian C. Schneider, Benita Schmitz‐Koep, Marcel Daamen, Lukas Scheef, Henning Boecker, Claus Zimmer, Nicole Baumann, Peter Bartmann, Dieter Wolke, Josef G. Bäuml, Christian Sorg

**Affiliations:** ^1^ Department of Neuroradiology Klinikum rechts der Isar, School of Medicine, Technical University of Munich Munich Germany; ^2^ Technical University of Munich‐NIC Neuroimaging Center Munich Germany; ^3^ Functional Neuroimaging Group, Department of Radiology University Hospital Bonn Bonn Germany; ^4^ Department of Neonatology University Hospital Bonn Bonn Germany; ^5^ Department of Psychology University of Warwick Coventry UK; ^6^ Warwick Medical School, University of Warwick Coventry UK; ^7^ Department of Psychiatry Klinikum rechts der Isar, School of Medicine, Technical University of Munich Munich Germany

**Keywords:** hippocampus, intelligence, magnetic resonance imaging, premature birth

## Abstract

Reduced global hippocampus volumes have been demonstrated in premature‐born individuals, from newborns to adults; however, it is unknown whether hippocampus subfield (HCSF) volumes are differentially affected by premature birth and how relevant they are for cognitive performance. To address these questions, we investigated magnetic resonance imaging (MRI)‐derived HCSF volumes in very premature‐born adults, and related them with general cognitive performance in adulthood. We assessed 103 very premature‐born (gestational age [GA] <32 weeks and/or birth weight <1,500 g) and 109 term‐born individuals with cognitive testing and structural MRI at 26 years of age. HCSFs were automatically segmented based on three‐dimensional T1‐ and T2‐weighted sequences and studied both individually and grouped into three functional units, namely hippocampus proper (HP), subicular complex (SC), and dentate gyrus (DG). Cognitive performance was measured using the Wechsler‐Adult‐Intelligence‐Scale (full‐scale intelligence quotient [FS‐IQ]) at 26 years. We observed bilateral volume reductions for almost all HCSF volumes in premature‐born adults and associations with GA and neonatal treatment intensity but not birth weight. Left‐sided HP, SC, and DG volumes were associated with adult FS‐IQ. Furthermore, left DG volume was a mediator of the association between GA and adult FS‐IQ in premature‐born individuals. Results demonstrate nonspecifically reduced HCSF volumes in premature‐born adults; but specific associations with cognitive outcome highlight the importance of the left DG. Data suggest that specific interventions toward hippocampus function might be promising to lower adverse cognitive effects of prematurity.

## Graphical abstract

Hedderich et al. report unspecific reductions of hippocampus subfield volumes in premature‐born adults. However, they found reduced dentate gyrus volumes to mediate the effect of low gestational age on adult full‐scale intelligence quotient suggesting functional relevance in premature‐born adults.

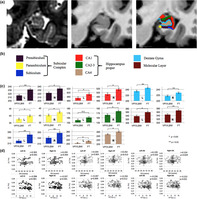

**Abbreviations**
ANOVA: analysis of variance
BLS: Bavarian longitudinal study
BW: birth weight
CA: cornu ammonis
CI: confidence interval
DG: dentate gyrus
FS‐IQ: full‐scale intelligence quotient
FT: full‐term
FWE: family‐wise error
FWHM: full‐width at half‐maximum
GA: gestational age
GC‐ML‐DG: granule cell and molecular layer of dentate gyrus
HC: hippocampus
HCSF: hippocampal subfield
HP: hippocampus proper
INTI: intensity of neonatal treatment index
MPRAGE: magnetization prepared rapid acquisition gradient echo
MRI: magnetic resonance imaging
MST: mnemonic similarity task
ROI: region of interest
SC: subicular complex
*SE*: standard error
SES: socioeconomic status
TE: time to echo
TI: time to inversion
TR: time to repetition
VLBW: very low birth weight
VP/VLBW: very preterm/very low birth‐weight
VP: very preterm
WAIS: Wechsler Adults Intelligence Scale

## 1 INTRODUCTION

Premature birth, that is, birth before 37 weeks of gestation, has a prevalence of around 11% worldwide (Chawanpaiboon et al., 2019; Howson, Kinney, McDougall, & Lawn, 2013). Brains of premature‐born newborns have shown to be particularly vulnerable to events of hypoxia‐ischemia, which is accompanied by an increased risk for extensive alterations of brain structure in newborns, children and adults, both on a microscopic (Back et al., 2002; Ball et al., 2013; Buser et al., 2012; Dean et al., 2013; Deng, 2010; Kinney et al., 2012; Salmaso, Jablonska, Scafidi, Vaccarino, & Gallo, 2014) and macroscopic level (Ball et al., 2012; Ball et al., 2014; Grothe et al., 2017; Meng et al., 2016; Ment, Hirtz, & Hüppi, 2009; Nosarti et al., 2008).

The hippocampus is a complex structure located in the medial temporal lobe, which is critical for neural pattern formation and its use in interaction with other structures, mainly cortices, for example in the context of memory consolidation, planning, recall, spatial navigation, or more complex concepts such as learning (Buzsaki & Moser, 2013). The function of the hippocampus as a “computational hub” is supported by its extensive connections to cortical and subcortical regions in the mammalian forebrain via the entorhinal and parahippocampal cortices (Sweatt, 2004). The hippocampus formation consists of structural and functional distinct subfields, most importantly the cornu ammonis (CA) subfields 1–4, the dentate gyrus (DG), which has an anatomical overlap with parts of CA4 and the subiculum (Amaral, 1978; Duvernoy, Cattin, Risold, Vannson, & Gaudron, 2013; Witter & Amaral, 2004). The human hippocampus is particularly vulnerable to hypoxic–ischemic events and its subfields exhibit differential vulnerability to hypoxia, ischemia, and hypercortisolemia (Bartsch et al., 2015; Sapolsky, 2000; Schmidt‐Kastner & Freund, 1991). For example, it was shown in human patients that the CA1 subfield is selectively vulnerable to a variety of metabolic and cytotoxic insults, in particular ischemia (Bartsch et al., 2015). The DG, as the primary site of adult neurogenesis in the hippocampal formation, has shown to be susceptible to increased stress and stress hormone levels (Schoenfeld & Gould, 2012). Volume reductions of distinct hippocampal subfields (HCSF) have been reported for several neuropsychiatric diseases such as major depressive disorder, posttraumatic stress disorder, and schizophrenia (Hayes et al., 2017; Nakahara, Matsumoto, & van Erp, 2018; Roddy et al., 2019) and also in patients after pediatric brain tumor surgery or microsurgical clipping of intracranial aneurysms (Decker et al., 2017; Hedderich, Reess, et al., 2019).

With respect to premature birth, lower whole hippocampus volumes have been described for infants, children, adolescents and adults and associations with memory functions were found (Aanes, Bjuland, Skranes, & Lohaugen, 2015; Bjuland, Rimol, Løhaugen, & Skranes, 2014; Lodygensky et al., 2008; Meng et al., 2016; Nosarti & Froudist‐Walsh, 2016). Further, in a cohort of premature‐born children at approximately 9 years of age, lower subfield volumes for the bilateral DG and the right subiculum as well as a positive correlation between right DG volume and working memory testing were reported (Aanes et al., 2019). However, the long‐term effect of premature birth on adult hippocampus structure has not been investigated, yet. Thus, it is not known whether lower hippocampus volumes after premature birth are due to selective vulnerability and volume loss of distinct subfields or whether the volume reduction is unspecific, probably because of general developmental impairment of the hippocampus. It is also unknown whether distinct HCSF volume reductions are specifically linked with cognitive function in adulthood after premature birth.

To address these questions, we analyzed HCSF in a large cohort of very premature‐born adults and term‐born controls using an established and automated pipeline based on T1‐ and T2‐weighted high‐resolution magnetic resonance imaging (MRI). We investigated associations with perinatal variables of premature birth and with adult full‐scale intelligence quotient (FS‐IQ) as a global outcome measure of cognitive performance in order to study their specificity for premature birth and their functional relevance. Furthermore, we investigated whether functional HCSF units mediate the relationship between gestational age (GA) and adult FS‐IQ in premature‐born adults.

## 2 MATERIALS AND METHODS

### 2.1 Participants

The participants examined in this study are part of the Bavarian Longitudinal Study (BLS), a geographically defined, whole‐population sample of neonatal at‐risk children and healthy full term controls who were followed from birth into adulthood (Riegel, Orth, Wolke, & Österlund, 1995; Wolke & Meyer, 1999). Of the initial 682 infants born very preterm (VP; <32 weeks) and/or with very low birth weight (VLBW) <1,500 g), 411 were eligible for the 26‐year follow‐up assessment, and 260 (63.3%) participated in psychological assessments (Breeman, Jaekel, Baumann, Bartmann, & Wolke, 2015). Of the initial 916 full‐term born infants from the same obstetric hospitals that were alive at 6 years, 350 were randomly selected as control subjects within the stratification variables of sex and family socioeconomic status (SES) in order to be comparable with the VP/VLBW group. Of these, 308 were eligible for the 26‐year follow‐up assessment, and 229 (74.4%) participated in psychological assessments. All of the 260 subjects from the VP/VLBW group underwent an initial screening for MR‐related exclusion criteria, which included: (self‐reported) claustrophobia, inability to lie still for >30 min, unstable medical conditions (e.g., severe asthma), epilepsy, tinnitus, pregnancy, nonremovable, MRI‐incompatible metal implants and a history of severe CNS trauma or disease that would impair further analysis of the data. The most frequent reason not to perform the MRI exam, however, was a lack of motivation. The remaining eligible, 103 VP/VLBW and 109 FT individuals underwent MRI at 26 years of age.

The MRI examinations took place at two sites: The Department of Neuroradiology, Klinikum rechts der Isar, Technical University of Munich (*n* = 144) and the Department of Radiology, University Hospital of Bonn (*n* = 68). The study was carried out in accordance with the Declaration of Helsinki and was approved by the local institutional review boards. Written consent was obtained from all participants. All study participants received travel expenses and a small payment for attendance. A more detailed description of participants, including incidental brain MRI findings can be found in a previous publication (Hedderich et al., 2020).

### 2.2 Birth‐related variables

GA was calculated from maternal reports on the first day of the last menstrual period and serial ultrasounds during pregnancy. In cases where the two measures differed by more than 2 weeks, clinical assessment at birth with the Dubowitz method was applied (Dubowitz, Dubowitz, & Goldberg, 1970). Maternal age, BW, and duration of hospitalization were obtained from obstetric records (Gutbrod, Wolke, Soehne, Ohrt, & Riegel, 2000; Riegel et al., 1995). Intensity of Neonatal Treatment Index (INTI) was determined by daily assessments of care level, respiratory support, feeding dependency and neurological status (mobility, muscle tone, and neurological excitability). Each of the six variables was scored on a 4‐point rating scale (0–3) by the method of Casaer and Eggermont (Casaer & Eggermont, 1985) (see Table S1 for a description of the variables). The INTI was computed as the mean score of daily ratings during the first 10 days of life or until a stable clinical state was reached (total daily scores <3 for 3 consecutive days), depending on which occurred first, ranging from 0 (best state) to 18 (worst state). Family SES was assessed through structured parental interviews within 10 days of childbirth. SES was computed as a weighted composite score based on the profession of the self‐identified head of each family together with the highest educational qualification held by either parent (Bauer, 1988).

### 2.3 Neurocognitive assessment

At 26 years of age, study participants were assessed using a short version of the German Wechsler Adults Intelligence Scale, Third edition (von Aster, Neubauer, & Horn, 2006): The assessment took place prior to and independent of the MRI scan and was carried out by trained psychologists who were blinded to group membership. Subsequently, an FS‐IQ was computed.

### 2.4 MRI data acquisition

MRI examinations were performed at both sites on either a Philips Achieva 3 T or a Philips Ingenia 3 T system using 8‐channel SENSE head‐coils. Subject distribution among scanners was as follows: Bonn Achieva 3 T: 5 VP/VLBW, 11 FT, Bonn Ingenia 3 T: 35 VP/VLBW, 17 FT, Munich Achieva 3 T: 60 VP/VLBW, 64 FT, Munich Ingenia 3 T: 3 VP/VLBW, 17 FT. To account for possible confounds by the scanner‐specific differences, all statistical analyses included categorical dummy regressors for scanner identity as covariates of no interest. Across all scanners, sequence parameters were kept identical. Scanners were checked regularly to provide optimal scanning conditions. MRI physicists at the University Hospital Bonn and Klinikum rechts der Isar regularly scanned imaging phantoms, to ensure within‐scanner signal stability over time. Signal‐to‐noise ratio was not significantly different between scanners (one‐way analysis of variance with factor “scanner‐ID” (Bonn 1, Bonn 2, Munich 1, Munich 2); *F*(3,182) = 1.84, *p* = .11). The image protocol included a high‐resolution T1‐weighted, 3D‐MPRAGE sequence (TI = 1,300 ms, TR = 7.7 ms, TE = 3.9 ms, flip angle 15°; acquisition matrix: 256 × 256) with a reconstructed isotropic voxel size of 1 mm^3^ and a high resolution T2‐weighted 3D sequence based on the variable refocusing flip angle technique (sweep technique) (Tschampa et al., 2015) (TR = 2,500 ms, TE = 364 ms, flip angle 90°; acquisition matrix: 256 × 256, reconstruction matrix: 512 × 512, echo train length: 120) with a reconstructed isotropic voxel size of 0.5 mm^3^. All images were visually inspected for artifacts.

### 2.5 HCSF volumetry

Image analysis was performed with the FreeSurfer image analysis suite (version 6.0), which is documented and freely available for download online (http://surfer.nmr.mgh.harvard.edu/) (Dale, Fischl, & Sereno, 1999; Fischl et al., 2002; Segonne et al., 2004). Within that framework, the cross‐sectional, multispectral pipeline was chosen and both T1‐weighted and T2‐weighted scans were used as input for HCSF volumetry. In this FreeSurfer release (v6.0), an HCSF segmentation tool has been implemented which is based on a Bayesian model with Markov random field priors (Iglesias et al., 2015). Briefly, the applied parametric segmentation algorithm was developed based on high‐resolution (0.13 mm) ex vivo MRI scans of the human hippocampus from 15 autopsy samples. These ex vivo MRI samples were manually segmented and integrated with in vivo T1‐weighted images (1‐mm resolution) in order to establish an atlas of the hippocampal formation with a new Bayesian inference algorithm to detect local variations in MRI contrast. The algorithm segments 12 different HCSFs, namely: hippocampal tail; presubiculum; parasubiculum; hippocampus‐amygdala‐transition‐area; molecular layer; granule cell and molecular layer of GC‐ML‐DG; fimbria; hippocampal fissure, CA (CA)1, CA2‐3, and CA4. For our study, we focused on core HCSF and built functional units: presubiculum, parasubiculum, and subiculum form the SC, CA1‐4 form the HP and GC‐ML‐DG is referred to as DG according to the classic anatomical literature and recent MRI studies of HCSFs (Roddy et al., 2019; Witter & Amaral, 2004). HCSF volumes of hippocampal tail, hippocampus‐amygdala‐transition‐area, fimbria, and hippocampal fissure were excluded. Output segmentations were inspected visually.

### 2.6 Statistical analysis

Statistical analyses were carried out using SPSS (IBM SPSS Statistics, version 25). General linear models were used to determine whether premature birth status is a significant factor for different HCSF volumes (dependent variable: HCSF; fixed factors: history of premature birth; covariates: sex, scanner, total intracranial volume [TIV]). This analysis was repeated using a similar general linear model, correcting for left/right hippocampus volumes instead of TIV. Partial correlations restricted to the VP/VLBW group and corrected for scanner, TIV, and sex were used to investigate the associations between HCSF volumes and variables of premature birth: GA, BW, and INTI. A similar partial correlation analysis was used to investigate FS‐IQ and HCSFs. Differences between VP/VLBW and FT individuals were tested using chi‐square tests (sex, SES) or two‐sample *t* tests (age, GA, BW, INTI, maternal age, FS‐IQ). Statistical significance was set at *p* < .05; all tests are two‐sided. *p*‐Values of post hoc tests were FDR‐corrected for multiple comparisons according to the Benjamini–Hochberg method within every discrete set of analyses (Benjamini & Hochberg, 1995). In order to test whether functional HCSF units mediate the association between low GA and FS‐IQ in adulthood, a mediation analysis restricted to the VP/VLBW group was performed using the PROCESS toolbox (version 3.0) (Hayes, 2017). In the mediation model, GA was entered as causal variable, adult FS‐IQ as the outcome variable, left and right HP, SC and DG volumes were introduced simultaneously as mediator variables, and MRI scanner, TIV and sex as covariates of no interest. Path coefficients for total effect, direct effect and indirect effect were estimated using (unstandardized) regression coefficients from multiple regression analyses, and statistical significance of the indirect effect was tested using a nonparametric bootstrap approach (with 5.000 repetitions) to obtain 95% confidence intervals. We calculated *p*‐values for indirect effects based on 95% confidence intervals, *SE* and estimated effect as described by Altman and Bland (Altman & Bland, 2011).

## 3 RESULTS

### 3.1 Sample characteristics

Group demographic and clinical background variables are shown in Table 1. Within the VP/VLBW group, 26 fulfilled only the VP criterion (GA < 32 weeks) and 21 fulfilled only the VLBW criterion (BW < 1,500 g), while 56 fulfilled both criteria. There were no significant differences between the VP/VLBW and FT group regarding age at scanning (*p* = .765), sex (*p* = .167), SES at birth (*p* = .492), and maternal age (*p* = .889). By design, VP/VLBW subjects had significantly lower GA (*p* < .001), lower BW (*p* < .001), neonatal treatment intensity as measured by INTI, and longer duration of hospitalization after birth (*p* < .001). TIV was significantly lower in VP/VLBW than in FT individuals (VP/VLBW: 1457.9 ± 141.1 mm^3^, FT: 1527.3 ± 149.3 mm^3^; *p* = .001). VP/VLBW subjects had lower FS‐IQ scores in adulthood (*p* < .001). For detailed results and group comparison of demographic, clinical, and cognitive data, please see Table 1.

**TABLE 1:** Demographic, clinical, and cognitive data

	VP/VLBW (*n* = 103)			FT (*n* = 109)			
	*M*	*SD*	Range	*M*	*SD*	Range	*p*‐Value
Sex (male/female)	59/44			64/45			.890
Age (years)	26.7	±0.61	25.7–28.3	26.8	±0.74	25.5–28.9	.170
GA (weeks)	30.5	±2.1	25–36	39.7	±1.1	37–42	<.001
BW (g)	1,318	±313	630–2070	3,392	±445	2,120–4,670	<.001
Hospitalization (days)	73	±26.9	24–170	7	±3.0	2–26	<.001
INTI	11.6	±3.7	3–18	n.a.	n.a.	n.a.	n.a.
SES^a^ (a.u.)	29/45/29		1–3	35/49/25		1–3	.651
Maternal age (years)	29.3	±4.7	16–41	29.4	±5.2	18–42	.937
FS‐IQ^b^ (a.u.)	94.2	±12.6	64–131	102.6	±12.0	77–130	<.001

*Note:* Statistical comparisons: sex, SES with *χ* ^2^ statistics; age, GA, BW, hospitalization, maternal age and FS‐IQ with two‐sample *t* tests.

Abbreviations: BW, birth weight; FS‐IQ, full‐scale intelligence quotient; FT, full‐term; GA, gestational age; INTI, intensity of neonatal treatment index; *M*, Mean; maternal age, maternal age at birth; *SD*, standard deviation; SES, socioeconomic status at birth; VP/VLBW, very preterm and/or very low birthweight.

^a^ 1 = upper class, 2 = middle class, 3 = lower class.

^b^ Data are based on 98 VP/VLBW and 106 FT‐born individuals.

### 3.2 Premature birth is associated with lower HCSF volumes in adulthood

**FIGURE 1:**
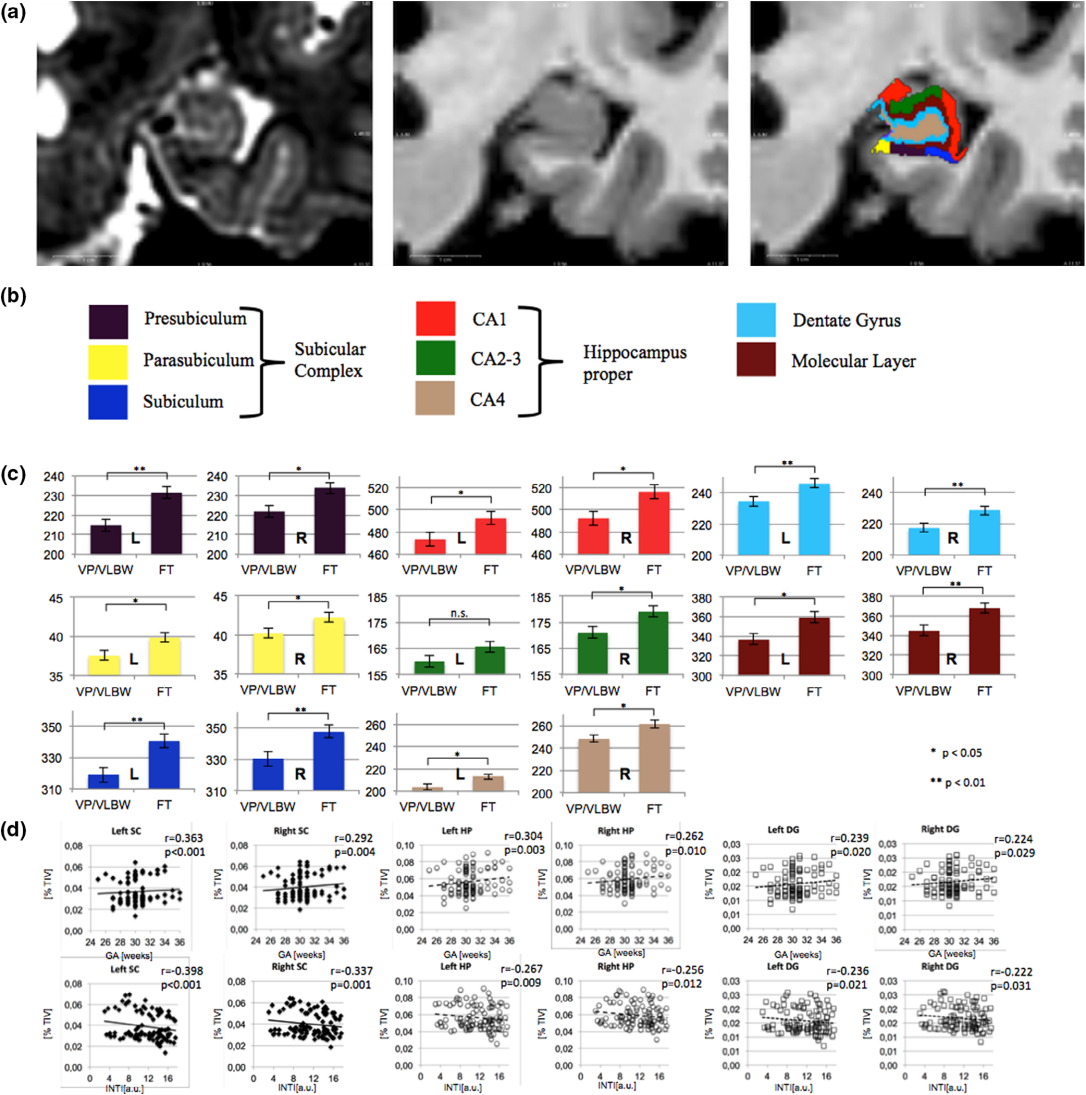
(a) Hippocampus subfield segmentation. Exemplary hippocampus subfield (HCSF) parcellation and labeling as produced by FreeSurfer v6.0 for a given individual's T2‐ and T1‐weighted input images are shown and depicted by a color legend (see below). Coronal slice inputs for the multispectral analysis are shown (left panel: T2‐weighted, middle panel: T1‐weighted). (b) Functional hippocampus units were formed as indicated: SC consisting of presubiculum, parasubiculum and subiculum and HP consisting of CA1‐4. (c) Lower hippocampus subfields in premature born adults. Marginal means of bilateral HCSF are shown as bar plots, error bars indicate *SE*. Group differences were assessed using a general linear model (fixed factor: prematurity at birth, covariates: sex, scanner, TIV). Colors correspond to the labels in Panel (a). Significant group differences after false discovery correction for multiple comparisons using the Benjamini–Hochberg method are marked with asterisks (*: *p* < .05; **: *p* < .01). (d) Associations between functional hippocampus units and variables of premature birth. Scatterplots of associations of left and right SC, HP, and DG are shown with GA (upper row) and intensity of neonatal treatment (INTI, lower row) are shown as scatterplots. Linear regression lines and regression coefficients of partial regression analyses are added. Functional hippocampus unit volumes are depicted as percentage of TIV. a.u., arbitrary units; CA, cornu ammonis; DG, dentate gyrus; FT, full‐term; GA, gestational age; HP, hippocampus proper; INTI, intensity of neonatal treatment; mm^3^, cubic millimeter; SC, subicular complex; TIV, total intracranial volume; VP/VLBW, very preterm and/or very low birthweight

Using a general linear model we found lower volumes in VP/VLBW adults for all investigated HCSF presubiculum, parasubiculum, subiculum CA1, CA2‐3, CA4, DG and molecular layer, after correction for scanner, sex, and TIV (see Figure 1b). Group differences were statistically significant for almost all HCSF volumes after correction for multiple testing, except left CA2‐3. Group differences of HCSF volumes were not significant if correcting for left or right whole hippocampus volumes instead of TIV in the general linear model (please see supplemental Table S2). We observed significantly lower whole hippocampus volumes on both sides. Left hippocampus: VP/VLBW: 2563.2 ± 28.7 mm^3^, FT: 2711.6 ± 27.6 mm^3^; *F* = 12.8, *p* < .001; right hippocampus: VP/VLBW: 2686.8 ± 29.3 mm^3^, FT: 2823.5 ± 28.1 mm^3^; *F* = 10.4, *p* = .001. At the level of functional units, we also observed significant group differences with lower HP and SC volumes in VP/VLBW individuals: left SC: VP/VLBW: 571.1 ± 7.7 mm^3^, FT: 612.2 ± 7.4 mm^3^; *p* < .001; left HP: VP/VLBW: 837.6 ± 10.2 mm^3^, FT: 871.1 ± 9.8 mm^3^; *p* = .025; right SC: VP/VLBW: 592.1 ± 7.7 mm^3^, FT: 623.9 ± 7.4 mm^3^; *p* = .005; right HP: VP/VLBW: 880.7 ± 10.8 mm^3^, FT: 923.6 ± 10.4 mm^3^; *p* = .007). For detailed information, please see Table 2.

**TABLE 2:** HCSF volumes

	VP/VLBW (*n* = 103)				FT (*n* = 109)				
	*M*	*SE*	95% CI		*M*	*SE*	95% CI		*p*‐Value
Left presubiculum (mm^3^)	214.7	3.1	208.5	220.8	231.6	3.0	225.7	237.5	**<.001**
Left parasubiculum (mm^3^)	37.6	0.6	36.4	38.8	39.9	0.6	38.7	41.0	**.011**
Left subiculum (mm^3^)	318.8	4.6	309.7	328.0	340.7	4.5	332.0	349.5	**.001**
Left CA1 (mm^3^)	473.6	6.0	461.7	485.4	492.4	5.8	481.0	503.8	**.032**
Left CA2‐3 (mm^3^)	160.0	2.2	155.7	164.3	165.6	2.1	161.5	169.8	.078
Left CA4 (mm^3^)	204.0	2.7	198.6	209.4	213.1	2.6	207.9	218.3	**.023**
Left DG (mm^3^)	234.4	3.0	228.5	240.3	245.8	2.9	240.1	251.5	**.009**
Left molecular layer (mm^3^)	337.2	5.9	325.5	349.0	359.2	5.7	347.9	370.4	**.011**
Right presubiculum (mm^3^)	221.7	3.1	215.6	227.9	233.8	3.0	227.9	239.7	**.008**
Right parasubiculum (mm^3^)	40.3	0.6	39.0	41.5	42.3	0.6	41.1	43.5	**.031**
Right subiculum (mm^3^)	330.1	4.5	321.3	339.0	347.8	4.3	339.3	356.3	**.007**
Right CA1 (mm^3^)	492.0	6.3	479.5	504.5	516.0	6.1	504.0	528.0	**.010**
Right CA2‐3 (mm^3^)	171.1	2.2	166.8	175.5	179.2	2.1	175.0	183.4	**.012**
Right CA4 (mm^3^)	248.5	3.1	242.4	254.7	261.2	3.0	255.3	267.1	**.005**
Right DG (mm^3^)	217.5	2.9	211.9	223.2	228.4	2.7	223.0	233.8	**.009**
Right molecular layer (mm^3^)	344.9	5.5	334.0	355.8	368.2	5.3	357.7	378.6	**.004**

*Note:* Marginal mean values of HCSF volumes are given in mm^3^. General linear model with prematurity status at birth as fixed factor. Scanner, sex, and TIV served as covariates of no interest. Post hoc comparisons are FDR‐corrected and significant *p*‐values are printed in bold.

Abbreviations: CA, cornu ammonis; CI, confidence interval; DG, dentate gyrus; FT, full‐term; HCSF, hippocampus subfield; *M*, mean; *SE*, standard error; VP/VLBW, very preterm and/or very low birthweight.

In order to strengthen the notion that HCSF volume reductions are related with prematurity, we investigated the associations between HCSF volumes and variables of premature birth (GA, BW, neonatal treatment intensity) using partial correlation analyses. Significant correlations, controlled for multiple testing, were observed with GA in 9 out of 16 HCSF volumes, most notably with the subiculum bilaterally and left CA1. No significant correlations were observed between BW and HCSF. High neonatal treatment intensity showed a significant, inverse association with 8 out of 16 HCSF, most notably bilateral subiculum and CA‐1. Detailed results are given in Table S3.

Investigating functional hippocampus units (i.e., composite units), we found significant correlations between all studied volumes, namely bilateral HP, SC and DG and GA and neonatal treatment intensity. Again, no correlations were observed with BW. For detailed results, please see Table 3.

**TABLE 3:** Hippocampus subfield volumes and variables of premature birth

	GA		BW		INTI	
	*r*	*p*‐Value	*r*	*p*‐Value	*r*	*p*‐Value
Left SC	.363	**<.001**	.088	.395	−.398	**<.001**
Left HP (CA fields)	.304	**.003**	.120	.246	−.267	**.009**
Left DG	.239	**.020**	.143	.166	−.236	**.021**
Right SC	.292	**.004**	.164	.113	−.337	**.001**
Right HP (CA fields)	.262	**.010**	.141	.173	−.256	**.012**
Right DG	.224	**.029**	.176	.087	−.222	**.031**

*Note:* Correlation coefficients from partial correlation analyses in the VP/VLBW sample are given. TIV, scanner, and sex served as covariates. Results were FDR‐corrected for multiple comparisons (18) using the Benjamini–Hochberg method and significant *p*‐values are printed in bold.

Abbreviations: BW, birth weight; CA, cornu ammonis; DG, dentate gyrus; FT, full‐term; GA, gestational age; HP, hippocampus proper; INTI, intensity of neonatal treatment; *M*, mean; SC, subicular complex; *SD*, standard deviation; TIV, total intracranial volume; VP/VLBW, very preterm and/or very low birthweight.

### 3.3 Left‐sided volume of the dentate mediates the impact of low GA on adult FS‐IQ in VP/VLBW individuals

In order to investigate the relevance of HCSF volume reductions for cognitive performance, we studied the association between altered HCSF volumes and adult FS‐IQ using correlation analyses. Significant positive associations, controlled for multiple testing, were found between left‐sided CA2‐3, CA4 and DG volumes and adult FS‐IQ. For detailed results, please see Table S4.

Investigating functional hippocampus units, we found significant correlations between left‐sided volumes, namely HP, SC, and DG volumes and adult FS‐IQ. No correlations were observed between volumes of right‐sided functional hippocampus units and FS‐IQ. For detailed results, please see Table 4.

**TABLE 4:** Hippocampus subfield volumes and cognitive function

	FS‐IQ	
	*r*	*p*‐Value
Left SC	.215	**.039**
Left HP (CA fields)	.282	**.006**
Left DG	.356	**<.001**
Right SC	.140	.184
Right HP (CA fields)	.145	.168
Right DG	.177	.091

*Note:* Correlation coefficients from partial correlation analyses in the VP/VLBW sample are given. TIV, scanner, and sex served as covariates. Results were FDR‐corrected for multiple comparisons (6) using the Benjamini–Hochberg method and significant *p*‐values are printed in bold.

Abbreviations: CA, cornu ammonis; DG, dentate gyrus; FS‐IQ, full‐scale intelligence quotient; HP, hippocampus proper; SC, subicular complex; TIV, total intracranial volume; VP/VLBW, very preterm and/or very low birthweight.

**FIGURE 2:**
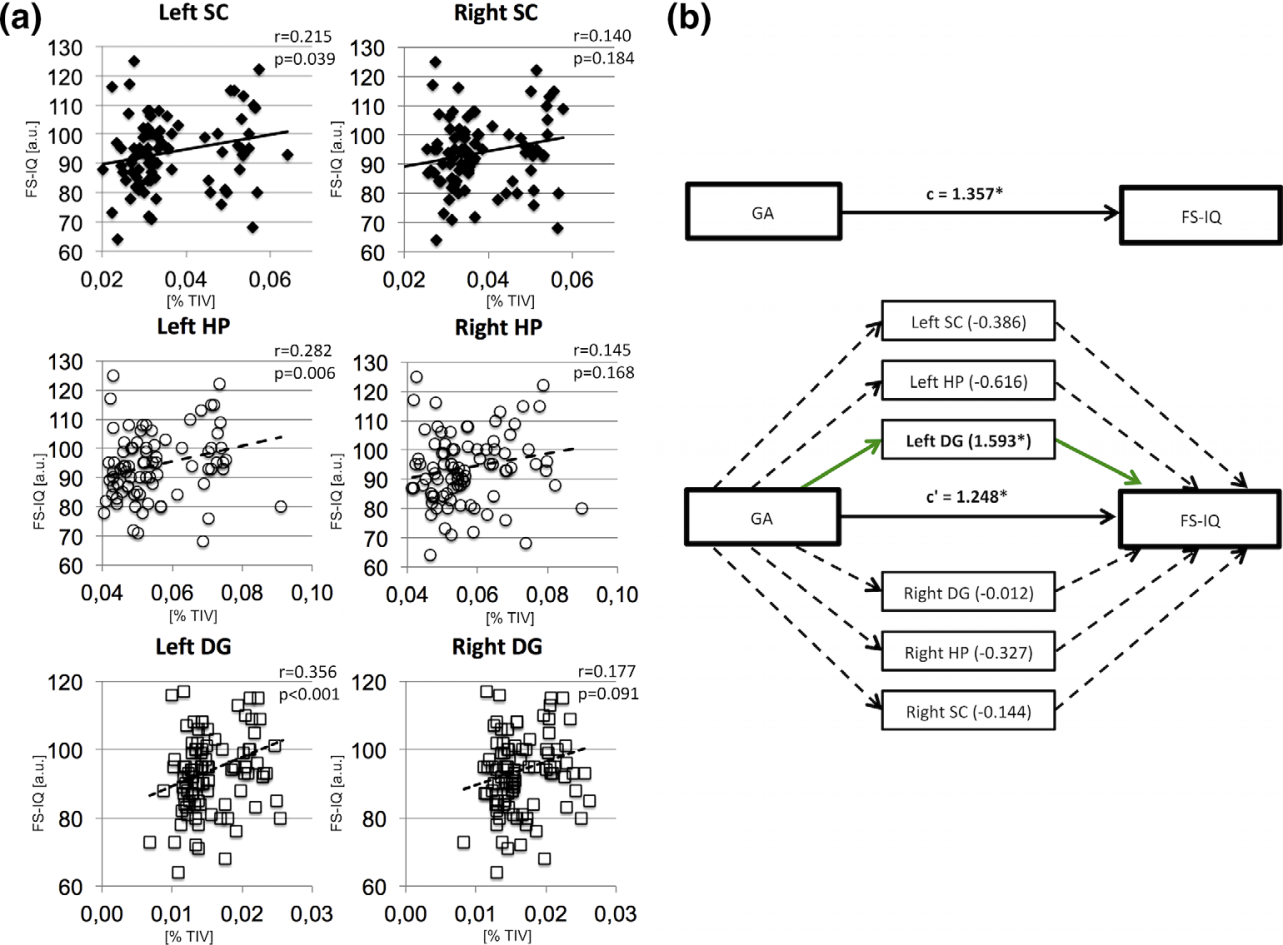
(a) Associations between functional hippocampus unit volumes and adult full‐scale intelligence quotient (FS‐IQ). Scatterplots of associations between left and right SC (upper row), HP (middle row), and DG (lower row) with adult FS‐IQ are shown. Linear regression lines and regression coefficients of partial regression analyses are added. Hippocampus subfield volumes are depicted as percentage of TIV. (b) Left DG volume is a mediator of global cognitive performance. A path diagram is shown in order to illustrate the result of the mediation analyses restricted to the VP/VLBW cohort. Gestational age significantly predicts adult FS‐IQ in the regression model correcting for sex, scanner, and TIV. Bilateral functional hippocampus units were introduced as mediators and left DG volume yielded a significant effect (1.593 ± 0.935; *p* = .044). The effect of GA on adult FS‐IQ remained significant after introduction of mediator variables. All other functional hippocampus units did not show significant mediating effects. The figure includes the following standardized regression coefficients: c, the total effect of GA on FS‐IQ; c′, the direct effect of GA on FS‐IQ when adjusting for the mediating variables. Significant regression coefficients (*p* < .05) are marked with an asterisk. DG, dentate gyrus; FS‐IQ, full‐scale intelligence quotient; HP, hippocampus proper; SC, subicular complex; TIV, total intracranial volume; VP/VLBW, very preterm and/or very low birth weight

We studied a possible effect of functional hippocampus units on the association between low GA and adult FS‐IQ by use of mediation analyses. We set low GA as causal variable, FS‐IQ as outcome variable and added six mediators in the same mediation model, namely left and right volumes of HP, SC and DG (see Figure 2b). In this mediation analysis, the total effect of low GA on adult FS‐IQ in the regression model was *c* = 1.357 ± 0.573; 95% CI: 0.220–2.494; *p* = .020. The direct effect of GA on adult FS‐IQ remained significant (*c*′ = 1.248 ± 0.576; 95% CI: 0.104–2.393; *p* = .033). The bootstrapped 95% CI determined that the indirect effect mediated by the left DG volume was significantly different from zero (ab = 1.593 ± 0.935; 95% CI: 0.203–3.790; *p* = .044), indicating a significant mediation effect on the association between GA and adult FS‐IQ. As determined by the 95% CIs, no significant indirect effect of the other functional hippocampus units included as mediators was observed. For detailed coefficients of indirect effects, please see Table S5.

## 4 DISCUSSION

Using MRI‐based HCSF in vivo volumetry, we demonstrated for the first time that all parts of the adult human hippocampus have lower volumes following a history of premature birth. We identified low GA and high neonatal treatment intensity to be associated with lower HCSF volumes after premature birth, but not low BW. By investigating the distinct functional hippocampus units SC, HP, and DG, we found that left DG mediated the effect of GA on adult cognitive performance.

### 4.1 Significantly lower HCSF volumes in adulthood after premature birth

We observed lower volumes for all HCSFs in adults after premature birth (Figure 1) after correction for TIV, sex, or scanner properties without any subfield‐specific volume reductions. This nonspecific reduction in HCSF volumes was supported by the fact that premature birth was not a significant factor in the general linear model correcting for left or right whole hippocampus volumes. Coming back to the initial question whether hippocampal volume reduction after premature birth is caused by subfield‐specific vulnerabilities or rather by an overall shifted developmental trajectory of the hippocampus, our results support the latter. However, it may also be possible that initial subfield‐specific volume reductions in earlier developmental stages were compensated by relative catch‐up growth, since there is robust evidence for subfield‐specific vulnerabilities toward conditions that are also associated with premature birth. For example, CA1 has been described as highly vulnerable to hypoxic–ischemic events, which are commonly associated with premature birth (Bartsch et al., 2015; Volpe, 2019). Moreover, there are studies from younger cohorts that found a more selective pattern of HCSF volume reductions: Aanes et al. reported that only the right subiculum volume and bilateral DG showed a significant group difference after correction for TIV (Aanes et al., 2019). A longitudinal study based on manual segmentation and shape analysis of the hippocampus found no significant difference of total hippocampal volume but localized subregional deformations in 15‐year‐old adolescents born preterm in the subiculum and CA1 subfields extending to CA2‐3 (Cole et al., 2015). On follow‐up, at around 19 years of age, these aberrations were mostly limited to subiculum and CA1 (Cole et al., 2015). In contrast, our results suggest a rather nonspecific long‐term effect of premature birth on bilateral HCSF volumes. We know from longitudinal MRI studies that HCSF volumes in fact grow uniformly, showing a steep volume increase until 13–15 years, followed by a phase with only small age‐related changes (Krogsrud et al., 2014). However, this growth pattern may be altered after premature birth by selectively injured HCSF that show catch‐up growth during late adolescence and early adulthood, while a global shift of the developmental brain trajectory persists.

### 4.2 Functional relevance of HCSF volumes for general cognitive performance

The hippocampus serves as a computational hub with vast connectivity to subcortical and cortical regions of the mammalian forebrain (Buzsaki, 2011; Sweatt, 2004). Specific hippocampal computational functions are sequential neural patterning including pattern completion and pattern separation (Buzsaki, 2011; Buzsaki & Moser, 2013). These are very basic processes needed for distinct cognitive functions such as planning, learning, and navigation, so that a central role for the development of general cognitive performance can be proposed. A MRI study in a rather small cohort of healthy individuals linked hippocampus volume with FS‐IQ in an inverse relationship, and reported that left‐sided hippocampus volume was associated with verbal IQ and right‐sided hippocampus volume with performance IQ (Amat et al., 2008). However, in the light of other studies investigating structural correlates of cognitive performance in the general population, it seems that—in case of an undamaged and well‐developed hippocampus—its volume is not sufficient to describe the variance in FS‐IQ scores (Gregory et al., 2016). The link between hippocampus and cognitive performance seems clearer in the context of premature birth, where prior evidence exists regarding the correlation of hippocampus structure and cognitive performance (Aanes et al., 2019; Sølsnes et al., 2015; Strahle et al., 2019; Thompson et al., 2013). In line with these findings, we found correlations between all left‐sided functional hippocampus units (i.e., SC, HP, and DG) and FS‐IQ. In contrast to these left‐sided findings, the associations between right‐sided functional hippocampus units and FS‐IQ were not statistically significant. This argues for a lateralization of hippocampus function in our cohort of VP/VLBW adults and is in line with a previous report on premature‐born young adults (Aanes et al., 2015). In this study, whole hippocampus volumes of the left side correlated with a higher number of memory test scores than right whole hippocampus volumes (Aanes et al., 2015). The differential pattern of significant correlations was explained by different functional properties of left and right hippocampi known from the general population with higher involvement of the left hippocampus in verbal and more general episodic memory, while the right hippocampus is more involved in spatial memory tasks (Burgess, Maguire, & O'Keefe, 2002; Suthana, Ekstrom, Moshirvaziri, Knowlton, & Bookheimer, 2011). This possible explanation may also apply to the lateralized finding in our study, assuming that typically “left hippocampal capacities” contribute more to general IQ than “right hippocampal capacities.” Another explanation for lateralized hippocampus function in our cohort may be that the left hippocampus is more severely affected by premature birth to a degree that volume loss becomes relevant for cognitive functioning. Although we observe bilateral hippocampal volume reductions, there is a trend toward increased volume reduction of the left hippocampus compared to the right side.

### 4.3 The DG mediates the impact of GA on adult FS‐IQ

It is known that premature‐born individuals are at increased risk for low cognitive performance throughout the life course which has been shown to be associated with several aspects of impaired brain development after premature birth (Ball et al., 2013; Hedderich, Bäuml, et al., 2019; Jaekel, Baumann, & Wolke, 2013; Lohaugen et al., 2010; Meng et al., 2016; Wolke, Johnson, & Mendonça, 2019). While we have observed unspecific volume reductions of HCSF after premature birth, functional associations were more complex. Explicitly, we found a specific mediation effect of the left DG volume on the relationship between low GA and adult FS‐IQ after premature birth. In contrast to our study, describing an unspecific reduction of virtually all HCSF, a recent study by Aanes et al. reported a more specific pattern of volume reductions including the bilateral DG in VLBW children (Aanes et al., 2019). However, our results indicate specific functional significance of the DG in the context of premature birth. The DG is a morphologically and functionally unique subfield of the human hippocampus. It is located adjacent to and partly coalesces with CA4 and contains a unique cell type, so‐called granule cells, which show very sparse firing activity and outnumber cells in other compartments such as the entorhinal cortex (Amaral, Scharfman, & Lavenex, 2007; Hasselmo, 2011). In computational models, the DG serves as a “pattern separator” by diversifying consolidated input from the entorhinal cortex (Hasselmo, 2011). Moreover, the DG is a site of adult neurogenesis, a fact that is thought to help its pattern separation task in the context of learning (Aimone, Deng, & Gage, 2010; Becker, 2005). Specific functional significance of the DG has been also shown in the context of aging by mediating the impact of aging on pattern separation performance (Dillon et al., 2017). Moreover, in patients with amnestic mild cognitive impairment, pharmacological regulation of DG activity improved cognitive performance at a memory task (Bakker et al., 2012). However, in our cohort, that includes young adults with impaired neurodevelopment, the impact of DG on FS‐IQ might be explained through an association with learning, since impaired learning processes might translate into lower global cognitive performance later on.

### 4.4 The hippocampus and its subfields—A possible target for therapeutic interventions?

In our opinion, the impact of the DG as a pattern separator and site of adult neurogenesis on the association between GA and FS‐IQ is of special importance. Given the multidimensional problem of impaired brain development, it is clear that HCSFs will only contribute a small part to the whole picture. Previous studies have found other structural brain aberrations correlating with adult cognitive performance, for example, global gray and white volumes or gyrification (Hedderich, Bäuml, et al., 2019; Nosarti et al., 2008). However, this differential contribution by HCSF is potentially significant since the hippocampus is involved in central cognitive‐behavioral functions of the brain. Furthermore, the hippocampus could be an attractive treatment target. First, its identification and volumetric measuring can be reliably performed across the lifespan, also in younger cohorts (Nosarti & Froudist‐Walsh, 2016). Second, we know that brains of premature‐born babies react to stimuli from the outside world, for example, stress, painful events or the level of language exposure on the neonatal intensive care unit (NICU). For example, it was shown that preterm babies treated in single‐patient rooms in the NICU had poorer language development, compared to preterm babies treated in an open‐ward setting, which was associated with abnormal folding of the left superior temporal cortex (Pineda et al., 2014). It can be assumed that a similar relationship exists between targeted treatment interventions, hippocampus structure, and functional outcomes. For example, it was shown that environmental enrichment (e.g., increasing stimuli for spatial navigation) had an effect on the hippocampus in mice that showed an increased number of neurons in the granule‐cell layer of the DG (Kempermann, Kuhn, & Gage, 1997). Also, learning of associative tasks (e.g., navigation in a water‐maze under different circumstances) which requires the hippocampus, increased adult neurogenesis in rats (Gould, Beylin, Tanapat, Reeves, & Shors, 1999). In humans, pattern separation, which is mainly carried out by the DG, can be tested explicitly by mnemonic similarity task (MST) which is used as a proxy for hippocampal integrity (Stark, Kirwan, & Stark, 2019). MST performance has been shown to improve with light aerobic exercise as well as through virtual environmental enrichment through specific exposure to 3D video games in young and older adults (Clemenson & Stark, 2015; Suwabe et al., 2017, 2018). Responsiveness of the human hippocampus to training interventions has been also demonstrated in humans using MRI‐based volumetry. Erickson et al. have shown that physical aerobic exercise in healthy older individuals was associated with an increase of hippocampal volume, increased levels of brain‐derived neurotrophic factor (which is thought to promote adult neurogenesis in the hippocampus), and improved performance in spatial short‐term memory tasks (Erickson et al., 2011). Another study found an effect of cognitive training on left hippocampus activation and performance during verbal memory tasks in patients with mild cognitive impairment (Rosen, Sugiura, Kramer, Whitfield‐Gabrieli, & Gabrieli, 2011). Taken together, it seems attractive to design treatment interventions targeted at hippocampus functions in premature‐born individuals, in order to promote their development.

### 4.5 Strengths and limitations

Some points should be carefully considered when interpreting our results. The current sample is biased to VP/VLBW adults with less severe neonatal complications, less functional impairments, and higher IQ. Individuals with stronger birth complications and/or severe lasting impairments in the initial BLS sample were more likely to be excluded for MRI screening due to specific MRI exclusion criteria (e.g., infantile cerebral palsy, the inability to lie still hypothetically due to underlying ADHD, or epilepsy) and to reject MRI assessment due to their stronger impairments and related lower level of general activity. Thus, differences in HCSF volumes between VP/VLBW and term‐born control adults reported here are conservative estimates of true differences. Classically, neurocognitive tests focusing on declarative memory are used in order to test hippocampus function clinically. However, no dedicated memory tests such as the MST were acquired in our study and we used FS‐IQ scores to correlate HCSF volumes with general cognitive performance. In our opinion, this is justified due to the large amount of hippocampus functions apart from memory encoding and retrieval such as spatial navigation or learning. It seems reasonable that especially impaired learning mechanisms might lead to impaired FS‐IQ in adulthood after premature birth. However, this rather indirect connection must be noted as a limitation. One of the strengths of our study is its large sample size (103 VP/VLBW and 109 FT adults), which enhances the generalizability of our findings. Moreover, the homogeneous and small age range of the study population in adulthood excludes effects of chronological age differences. Another strength of our study is that high‐quality brain imaging was performed so that both high‐resolution T1‐weighted and T2‐weighted input images could be used for HCSF segmentation. Advances in automated segmentation pipelines have made this study possible because the high workload of manual delineations severely limits sample size in brain imaging studies. However, several concurrent automated segmentation protocols have been published with slight differences with regard to subfield definition (Mueller et al., 2018).

### 4.6 Conclusion

Our results show a general reduction of HCSF volumes in adulthood after premature birth, associated with low GA and high neonatal treatment intensity, not BW. We propose a potential dominating effect of a shifted developmental trajectory after premature birth over a special vulnerability of HCSF. Moreover, we demonstrated a significant mediation effect of left DG on the relationship between low GA and adult FS‐IQ as well as for long‐term cognitive development, underlining the functional relevance of this HCSF that is important for pattern separation and adult neurogenesis. In our opinion, this underlines that the hippocampus may constitute a potential treatment target for interventions aiming at improving long‐term cognitive outcomes after premature birth. Future studies will be needed in order to integrate several modules of impaired brain development after premature birth into a more complete framework with respect to social, biological, and psychological factors.

## CONFLICT OF INTEREST

The authors declare no conflict of interest.

## Supporting information


**Appendix** **S1**: Supporting Information

## Data Availability

Patient data used in this study are not publicly available but stored by the principal investigators of the Bavarian Longitudinal Study.
