# Supplementary material for: Hippocampal subfield volumes are nonspecifically reduced in premature‐born adults
Source: Hum Brain Mapp. 2020 Aug 26;41(18):5215–27. doi: 10.1002/hbm.25187 (PMC7670635; doi:10.1002/hbm.25187)
Supplement: Supplementary file 1 — Appendix S1: Supporting Information [file HBM-41-5215-s001.docx]

**Table S1: Description of the variables comprising the INTI**

| **Scale value** | **Scale label** |
| --- | --- |
| 1. **Care level** | |
| 0 | Normal care (i.e. that required by term-born infant) |
| 1 | Special care |
| 2 | Intensive observation |
| 3 | Intensive treatment |
| 1. **Respiratory support** | |
| 0 | Air |
| 1 | Extra oxygen |
| 2 | CPAP |
| 3 | Ventilator |
| 1. **Feeding dependency** | |
| 0 | Oral |
| 1 | Gavage/ probe |
| 2 | Intravenous plus gavage and/or oral |
| 3 | Intravenous |
| 1. **Mobility** | |
| 0 | Normal |
| 1 | No label specified |
| 2 | Slightly diminished or elevated |
| 3 | Extremely diminished or elevated (hyper or hypokinetic) |
| 1. **Muscle tone** | |
| 0 | Normal |
| 1 | No label specified |
| 2 | Slightly degraded or elevated |
| 3 | Hypertonic or hypotonic |
| 1. **Neurological excitability (of the Central Nervous System)** | |
| 0 | Normal |
| 1 | No label specified |
| 2 | Slightly delayed / weak or increased reactions possibly with trembling |
| 3 | Pronounced, Decreased (apathy, coma) or severe hyperexcitability with abnormal signs |

Description of the six variables that comprise the INTI.

Abbreviations: CPAP, continuous positive airway pressure; INTI, intensity of neonatal treatment index.

**Table S2: Hippocampus subfield volumes corrected for left/right whole hippocampus volumes**

|  | VP/VLBW (n=103) | | | | FT (n=109) | | | |  |
| --- | --- | --- | --- | --- | --- | --- | --- | --- | --- |
|  | M | SE | 95%-CI | | M | SE | 95%-CI | | p value |
| Left Presubiculum [mm^3^] | 221.7 | 2.1 | 217.7 | 225.8 | 226.9 | 2.0 | 222.9 | 230.7 | 0.091 |
| Left Parasubiculum [mm^3^] | 38.8 | 0.5 | 37.9 | 39.8 | 39.1 | 0.5 | 38.2 | 40.1 | 0.669 |
| Left Subiculum [mm^3^] | 331.4 | 2.4 | 326.6 | 336.1 | 332.5 | 2.3 | 327.9 | 337.1 | 0.741 |
| Left CA1 [mm^3^] | 488.8 | 2.7 | 483.5 | 494.0 | 480.5 | 2.6 | 475.4 | 585.6 | 0.034 |
| Left CA2-3 [mm^3^] | 165.5 | 1.4 | 162.8 | 168.1 | 161.7 | 1.3 | 159.1 | 164.3 | 0.063 |
| Left CA4 [mm^3^] | 211.5 | 1.3 | 209.0 | 214.1 | 207.7 | 1.3 | 205.2 | 210.3 | 0.048 |
| Left DG [mm^3^] | 242.7 | 1.3 | 240.2 | 245.3 | 239.9 | 1.2 | 237.4 | 242.3 | 0.129 |
| Left Molecular Layer [mm^3^] | 347.7 | 4.6 | 338.6 | 356.8 | 348.8 | 4.5 | 340.0 | 357.6 | 0.872 |
| Right Presubiculum [mm^3^] | 228.5 | 2.1 | 224.5 | 232.6 | 229.3 | 2.0 | 225.4 | 233.3 | 0.780 |
| Right Parasubiculum [mm^3^] | 41.3 | 0.5 | 40.4 | 42.3 | 41.6 | 0.5 | 40.7 | 42.6 | 0.672 |
| Right Subiculum [mm^3^] | 341.1 | 2.3 | 336.6 | 345.6 | 340.3 | 2.2 | 335.9 | 344.6 | 0.793 |
| Right CA1 [mm^3^] | 508.0 | 2.3 | 503.5 | 512.5 | 503.5 | 2.3 | 499.1 | 507.9 | 0.183 |
| Right CA2-3 [mm^3^] | 176.0 | 1.4 | 173.2 | 178.8 | 175.9 | 1.4 | 173.2 | 178.6 | 0.958 |
| Right CA4 [mm^3^] | 224.4 | 1.4 | 221.7 | 227.1 | 223.5 | 1.3 | 220.9 | 226.2 | 0.665 |
| Right DG [mm^3^] | 256.0 | 1.3 | 253.4 | 258.6 | 255.6 | 1.3 | 253.1 | 258.2 | 0.837 |
| Right Molecular Layer [mm^3^] | 352.1 | 4.2 | 343.8 | 360.5 | 360.1 | 4.1 | 352.0 | 368.2 | 0.199 |

Marginal mean values of hippocampus subfield volumes are given in mm^3^. General linear model with prematurity status at birth as fixed factor. Scanner, sex and left/right whole hippocampus volume served as covariates of no interest. Post-hoc comparisons are FDR-corrected and significant p-values are printed in bold.

Abbreviations: CA, cornu ammonis; CI, confidence interval; DG, dentate gyrus; FT, full-term; M, mean; SE, standard error; VP/VLBW, very preterm and/or very low birthweight.

**Table S3: Hippocampus subfield volumes and variables of premature birth**

|  | Side | GA | | BW | | INTI | |
| --- | --- | --- | --- | --- | --- | --- | --- |
|  |  | r | p value | r | p value | r | p value |
| Presubiculum | L | 0.314 | **0.002** | 0.072 | 0.488 | -0.347 | **0.001** |
| Parasubiculum | L | 0.174 | 0.091 | 0.011 | 0.915 | -0.297 | **0.003** |
| Subiculum | L | 0.383 | **<0.001** | 0.100 | 0.337 | -0.405 | **<0.001** |
| CA1 | L | 0.342 | **0.001** | 0.090 | 0.388 | -0.308 | **0.002** |
| CA2-3 | L | 0.253 | **0.013** | 0.133 | 0.200 | -0.172 | 0.096 |
| CA4 | L | 0.205 | 0.046 | 0.158 | 0.127 | -0.202 | 0.050 |
| DG | L | 0.239 | 0.020 | 0.143 | 0.166 | -0.236 | 0.021 |
| Molecular Layer | L | 0.249 | **0.015** | 0.159 | 0.125 | -0.313 | **0.002** |
| Presubiculum | R | 0.260 | **0.011** | 0.136 | 0.189 | -0.259 | **0.011** |
| Parasubiculum | R | 0.071 | 0.494 | 0.022 | 0.830 | -0.204 | 0.048 |
| Subiculum | R | 0.310 | **0.002** | 0.145 | 0.162 | -0.368 | **<0.001** |
| CA1 | R | 0.271 | **0.008** | 0.137 | 0.185 | -0.289 | **0.004** |
| CA2-3 | R | 0.268 | **0.009** | 0.240 | 0.019 | -0.197 | 0.056 |
| CA4 | R | 0.206 | 0.045 | 0.143 | 0.167 | -0.200 | 0.052 |
| DG | R | 0.224 | 0.029 | 0.176 | 0.087 | -0.222 | 0.031 |
| Molecular Layer | R | 0.070 | 0.499 | 0.143 | 0.165 | -0.191 | 0.063 |

Correlation coefficients from partial correlation analyses in the VP/VLBW sample are given. TIV, scanner and sex served as covariates. Results were FDR-corrected for multiple comparisons (48) using the Benjamini-Hochberg method and significant p-values are printed in bold.

Abbreviations: BW, birth weight; CA, cornu ammonis; DG, dentate gyrus; FT, full-term; GA, gestational age; INTI, intensity of neonatal treatment; L, left; M, mean; R, right; SD, standard deviation; TIV, total intracranial volume; VP/VLBW, very preterm and/or very low birthweight.

**Table S4: Hippocampus subfield volumes and adult full-scale IQ**

|  | Side | FS-IQ | |
| --- | --- | --- | --- |
|  |  | r | p value |
| Presubiculum | L | 0.225 | 0.031 |
| Parasubiculum | L | 0.204 | 0.051 |
| Subiculum | L | 0.189 | 0.072 |
| CA1 | L | 0.207 | 0.048 |
| CA2-3 | L | 0.331 | **0.001** |
| CA4 | L | 0.360 | **<0.001** |
| DG | L | 0.356 | **<0.001** |
| Molecular Layer | L | 0.171 | 0.102 |
| Presubiculum | R | 0.126 | 0.232 |
| Parasubiculum | R | 0.194 | 0.064 |
| Subiculum | R | 0.127 | 0.228 |
| CA1 | R | 0.119 | 0.260 |
| CA2-3 | R | 0.183 | 0.080 |
| CA4 | R | 0.156 | 0.136 |
| DG | R | 0.177 | 0.091 |
| Molecular Layer | R | 0.139 | 0.186 |

Correlation coefficients from partial correlation analyses in the VP/VLBW sample are given. TIV, scanner and sex served as covariates. Results were FDR-corrected for multiple comparisons (48) using the Benjamini-Hochberg method and significant p-values are printed in bold.

Abbreviations: CA, cornu ammonis; DG, dentate gyrus; FS-IQ, full-scale intelligence quotient; FT, full-term; L, left; M, mean; R, right; SD, standard deviation; TIV, total intracranial volume; VP/VLBW, very preterm and/or very low birthweight.

**Table S5: Mediation analysis: Gestational age, functional hippocampus unit volumes and adult full-scale IQ**

| Functional unit | Side | Effect | SE | Lower 95%-CI | Upper 95%-CI | p value |
| --- | --- | --- | --- | --- | --- | --- |
| SC | L | -0.3860 | 0.5196 | -1.5246 | 0.5954 | 0.677 |
| HP | L | -0.6160 | 0.9009 | -2.6198 | 0.9661 | 0.672 |
| DG | L | 1.5926 | 0.9347 | 0.2032 | 3.7901 | **0.044** |
| SC | R | -0.1436 | 0.4729 | -1.1529 | 0.7791 | 0.598 |
| HP | R | -0.3266 | 0.9833 | -2.2434 | 1.7826 | 0.606 |
| DG | R | -0.0119 | 0.892 | -2.0623 | 1.6588 | 0.505 |

Indirect effects and standard errors of all included functional hippocampus unit included in the mediation analysis in the VP/VLBW sample are given. TIV, scanner and sex served as covariates. Significant p-values are printed in bold.

Abbreviations: CI, confidence interval; DG, dentate gyrus; HP, hippocampus prorper; IQ, intelligence quotient; L, left; R, right; SE, standard error; TIV, total intracranial volume; VP/VLBW, very preterm and/or very low birthweight.
